# A Nutraceutical Strategy to Slowing Down the Progression of Cone Death in an Animal Model of Retinitis Pigmentosa

**DOI:** 10.3389/fnins.2019.00461

**Published:** 2019-05-17

**Authors:** Ilaria Piano, Vanessa D’Antongiovanni, Lara Testai, Vincenzo Calderone, Claudia Gargini

**Affiliations:** ^1^Department of Pharmacy, University of Pisa, Pisa, Italy; ^2^Department of Clinical and Experimental Medicine, University of Pisa, Pisa, Italy

**Keywords:** retinitis pigmentosa, photoreceptors, retinal degeneration, oxidative stress, nutraceutical treatment

## Abstract

Retinitis pigmentosa (RP) is an inherited retinal dystrophy characterized by progressive degeneration of the visual cells and abnormalities in retinal pigment epithelium, the vision is lost slowly, and the final outcome is total blindness. RP primarily affects rods, but cones can also be affected as a secondary effect. Photoreceptor cell death is usually triggered by apoptosis, however the molecular mechanisms linking the rod degeneration to the secondary cone death are poorly understood. Possible causes of the secondary cone death are oxidative stress and/ or the release of toxic factors from dying rods. The aim of this study is to analyze the effect of nutraceutical molecules with antioxidant properties, on the progression of the disease in an established animal model of RP, and rd10 mice. We show that chronic treatment *per os* with a flavanone (naringenin) or a flavonol (quercetin) present in citrus fruits, grapes and apples, preserves retinal morphology, and ameliorates functionality. These actions are associated with a significant reduction of stress-oxidative markers, such as the detoxifying enzymes Sod1 and Sod2. In addition, naringenin and quercetin treatment reduces the levels of acrolein staining associated with a reduction of ROS in the cellular environment. The study demonstrates the beneficial effects of naringenin and quercetin, two molecules that possess antioxidant properties, limiting neurodegeneration, and thus preventing cone damage.

## Introduction

Retinitis pigmentosa (RP) is a group of inherited retinal diseases in which a mutation causes the death of rod photoreceptors. As a consequence, scotopic vision is impaired and the visual field is reduced as compared to healthy subjects (Wang et al., 2005). Subsequently, cones gradually die, and daily vision is lost. RP-associated genes are mainly expressed in rods and play a critical role in rod function and survival. In humans and in the animal models of this disease, such as rd1 (Lin et al., 2009) and rd10 mice (Gargini et al., 2007) cone photoreceptors are spared as long as a significant fraction of rod survives, suggesting a link between rod survival, and cone degeneration (Piano et al., 2013). Thus, while genetic mutations are the primary cause of the rod degeneration, cone death is possibly the consequence of the release of toxic substances, either released by a dying rod into the retinal milieu, or diffused to coupled cones through gap junctions (Ripps, 2002). Additionally, the lack of factors secreted by rods that are essential to the cone viability may lead to cone death (Roque et al., 1996). Furthermore, it has been suggested that the migration of microglial cells into the outer retina could contribute to photoreceptor degeneration in RP (Leveillard et al., 2004; Peng et al., 2014). Other evidence suggests that an upregulation of genes implicated in cellular metabolism may occur at various stages of the disease. As an example, the upregulation of the genes involved in the insulin/mTOR pathway supports the notion that metabolic stress is a contributing factor to the secondary death of cones (Punzo et al., 2009; Venkatesh et al., 2015). Emerging studies show that all these factors contribute to making cones in RD retinas much more sensitive to oxidative stress than their counterpart in healthy retinas, thus triggering processes of cell death (Campochiaro and Mir, 2018).

Oxidative stress involves the generation of free radicals which are inherently unstable, leading to oxidization of molecules in the local environment. Small amounts of ROS, generated by NADPH oxidase inside the cytoplasm, or by oxygen-electron donor mismatches present in mitochondria, are neutralized by glutathione and superoxide dismutase family enzymes (Sod). However, under pathological conditions, such as high tissue levels of oxygen or other sources of free radical generators, the antioxidant system may be insufficient to clear the environment from ROS, leading to the production of radicals even more harmful to the cell such as hydroxyl radicals. When free radicals encounter macromolecules, they produce characteristic modifications that compromise lipids, proteins, and DNA constituting oxidative damage (Campochiaro and Mir, 2018).

In the mouse retina, rods constitute 97.2% of the cells in the ONL and they are packed with mitochondria making them highly metabolically active (Jeon et al., 1998). As a result of rod death, the levels of oxygen consumption dramatically drop, leading to an increased oxidative stress that, in a second phase of the pathology, and contributes to the death of cones. The hypothesis that oxidative stress is the initial cause of cone degeneration is also supported by previous studies showing that the use of the anti-oxidant mixture is effective in slowing cones death down in different RP models, including rd1, Q344ter, rd10 mice (Komeima et al., 2007, 2008; Oveson et al., 2011), and P23H rats (Fernandez-Sanchez et al., 2012).

Here we show that two flavonoids present in a typical Western diet are effective in preventing the death of the cone photoreceptors in the animal model of RP, rd10 mice. These are quercetin, one of the most often studied dietary flavonoids present in various vegetables, tea, and red wine (D’Andrea, 2015) which interacts with misfolded rhodopsin molecules (Herrera-Hernandez et al., 2017) and naringenin, a bioflavonoid compound present in high concentrations in the Citrus species (Viswanatha et al., 2017).

## Materials and Methods

### Animals

rd10 mice were used as model of retinal neurodegeneration. These mice carry a homozygous phosphodiesterase 6b mutation (Pde6b^rd10/rd10^) on a C57Bl/6J background. Progressive rod, and then cone, photoreceptors degeneration in homozygous rd10 mice begins at postnatal day (P)18 and completes at day 60 (Gargini et al., 2007). Mice were kept at a constant room temperature with a light/dark cycle of 12 h with illumination levels below 60 lux. Animals were treated in accordance with Italian and European institutional guidelines, following experimental protocols approved by the Italian Ministry of Health (Protocol #DGSAF0001996/2014, Department of Pharmacy, University of Pisa, Pisa, Italy) and by the Ethical Committees of University of Pisa. For all experiments the animals were deeply anesthetized by an intraperitoneal injection of Urethane 20% in a saline buffer (0.9% NaCl) at dose of 0.1 ml/10 g and then killed by cervical dislocation under deep anesthesia. Age-matched C57Bl/6J mice were used as a wild type (wt) control.

### Nutraceutical Treatments

rd10 mice were used in a range of ages from P18 (when they are able to drink on their own) to P45 (peak of death of cone photoreceptors). Stock solutions of naringenin and quercetin were prepared in DMSO and added to their drinking water, resulting in a dose of 100 mg/kg/die *per* animal (Alam et al., 2013; Testai et al., 2013; Viswanatha et al., 2017). The water intake *per* mouse was measured daily to ensure each animal received the administered dose. The animals were randomly divided into three treatment groups as follows: (1) the vehicle group (*n* = 6) which received the same percentage of vehicle present in the water of the other groups (0,025% DMSO *per os*); (2) naringenin group (*n* = 6) which received 100 mg/kg/die; and (3) quercetin group (*n* = 6) treated with 100 mg/kg/die.

### Electroretinogram

The general procedure for animal preparation, anesthesia, ERG recording, light stimulation, and data analysis has been described in detail previously (Della Santina et al., 2012). ERGs were recorded in complete darkness via coiled gold electrodes making contact with the moist cornea. A small gold needle placed in the scalp served as both the reference and ground. Responses were amplified differentially, band-pass filtered at 0.1–500 Hz, digitized at 12.8 kHz by a computer interface (LabVIEW 6.1; National Instruments, Austin, TX, United States) and stored on a disk for processing. Light stimuli were delivered into a Ganzfeld sphere of 30 cm in diameter, with the internal surface coated with highly reactive white paint, to ensure uniform illumination of the whole retinal surface. For the flash stimulation protocol, an electronic flash unit (Sunpak B3600 DX, Tocad Ltd, Tokyo, Japan) delivered flashes of white light, the energy of which decayed with a t of 1.7 ms. Calibrated neutral density filters were used to attenuate the intensity of the flashes. Mice were subjected to scotopic and photopic ERG recordings. For scotopic ERG recordings, mice were subjected to different flash intensities (ranging from 1.71 × 10^–5^ to 377.2 cd*s/m^2^ in steps of 0.6 log units), each repeated five times, with an inter-stimulus interval that ranged from 20 s for dim flashes to 1 min for the brightest flashes. Five ERG traces at each flash luminance were averaged before measurements of b-wave amplitudes. Isolated cone components were obtained by superimposing the test flashes of eight different intensities (ranging from 0.016 to 377.2 cd*s/m^2^) on a steady rod-saturating background (30 cd/m^2^) after at least 15 min from background onset. Each light stimulus was repeated five times, with an inter-stimulus interval that ranged from 20 s for dim flashes to 1 min for the brightest flashes. Five ERG traces at each flash luminance were averaged before measurements of b-wave amplitudes and were analyzed offline using custom-compiled programs developed by LabView 7 (National Instruments).

### Western Blot

Retinas from C57Bl/6J and rd10 mice were lysed in modified RIPA buffer as described by Piano et al. (2013) and protein was quantified with the Bradford assay (Bio-Rad). For every experiment performed, three different samples from each experimental group were loaded on the gel, each from an animal previously used for ERG recordings. Proteins (25 μg) were separated onto a pre-cast 4–20% polyacrylamide gel (Mini-PROTEAN^®^ TGX gel, Bio-Rad) and transferred to PVDF membranes (Trans-Blot^®^ Turbo^TM^ PVDF Transfer packs, Bio-Rad). Membranes were blocked with 5% BSA diluted in Tris–buffered saline (TBS, 20 mM Tris–HCl, PH 7.5, 150 mM NaCl) with 0.1% Tween 20. Primary antibodies (Table 1) were incubated overnight at 4°C. After 3 × 10 min wash in T-TBS (TBS buffer with 0.1% of Tween-20), membranes were incubated with secondary antibodies (Table 1) for 2 h at room temperature. The immunoblot signal was visualized using an enhanced chemiluminescence substrate detection system (Luminata^TM^ Forte Western HRP Substrate, Millipore). The chemiluminescent images were acquired by LAS4010 (GE Healthcare Life-Sciences, Pittsburgh, PA, United States). Densitometry was undertaken using ImageJ software.

**TABLE 1:** List of antibodies.

Antibody	Company	Work dilution	Application
Actin	Merck Millipore	1:5000	WB
ATP5a	AbCam	1:500	WB
Cone-opsin R/G	Santa Cruz	1:1000	WB
Cone-opsin B	Santa Cruz	1:1000	WB
Sod1	Sigma Aldrich	1:500	WB
Sod2	Sigma Aldrich	1:500	WB
Anti-mouse IgG HRP conjugated	Merck Millipore	1:5000	WB
Anti-rabbit IgG HRP conjugated	Cell Signaling Technology	1:5000	WB

*WB, western blot analysis.*

Each protein of interest to the study was normalized for the content of a specific reference protein (β-actin or ATP5a). The reference protein was measured on the same membrane after a stripping procedure performed with three washes of 10 min each in glycine buffer pH 2, followed by three washes in T-TBS and blocking in 5% BSA. The incubation of primary and secondary antibodies (Table 1) was performed with the same protocol described above.

### Immunohistochemistry

The survival rate of the cone and the lipid peroxidation, as an indication of oxidative stress, were examined immunohistochemically. Frozen retinal sections (14 μm) were washed 3 × 10 min, in PBS, then incubated for 45 min in blocking solution (1% BSA, 0.3% Triton-X100 in PBS). Sections were then incubated with anti-cone arrestin antibody (1:5000, Merck Millipore) or anti-acrolein antibody (1:1000, AbCam) overnight at 4°C. After being washed 3 × 10 min in PBS, sections were incubated with the secondary antibody (goat anti-rabbit Alexa Fluor^®^ 488, Molecular Probe) for 2 h at room temperature. Sections were then washed 3 × 10 min in PBS and then incubated with a solution of ethidium homodimer diluted in PBS (1:5000, Sigma-Aldrich) for 5 min and washed once with PBS. Finally, slides were covered with a mounting medium (Vectashild^®^). Images were obtained with a Leica TCS-SP5 confocal microscope, using a 20× and 40× oil objective (0.75 and 1.45 NA, respectively) and a pinhole size of <1.0 μm. Scanning fields were 250 μm × 250 μm × 10 μm. Saved files were extended focus images obtained automatically by superposition of the five focal planes.

### Statistical Analysis

Statistical comparisons for ERG and western blot analysis were performed with analysis of variance (ANOVA) one-way or two-way test followed by Bonferroni-corrected *t*-test using Origin Lab 8.0 software (Microcal, Northampton, MA, United States).

## Results

### Functional and Morphological Retinal Recovery in rd10 Mice After Nutraceutical Treatment

**FIGURE 1:**
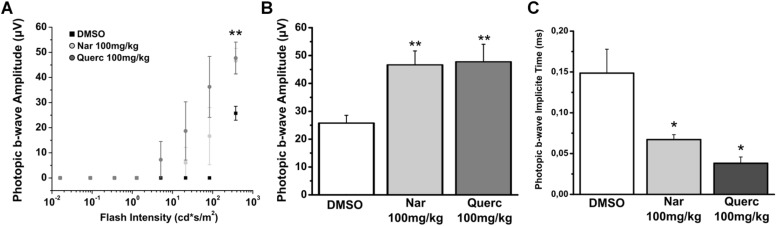
Naringenin and quercetin slow down the decline of photoreceptor function in rd10 mice. Photopic ERGs were performed at P45. **(A)** Sensitive curve of amplitude of photopic ERG response in function of increase intensity of light stimulus. **(B)** Histograms represent the maximum b-wave amplitude in response to the brightest flash. The bars are expressed as average ± SEM. **(C)** Histograms represent the implicit time of the maximum amplitude in response to the brightest flash. The dots are expressed as average ± SEM. Statistical analysis is one-way ANOVA followed by Bonferroni’s multiple comparison test (*p ≤ 0.05; **p ≤ 0.01). Number of animals for each treatment group is *n* = 6.

The results in this study show that chronic oral treatment with molecules of natural origin slows down the loss of functionality of the cone pathway and is effective in maintaining the morphological structure of these photoreceptors. However, the treatment fails to maintain any ERG response to the scotopic light stimuli (data not shown). Conversely the photopic b-wave is maintained in treated mice, as shown in Figure 1. Figure 1A shows the ERG response of the three treatment groups, the b-wave amplitude is plotted for the response to photopic flashes of different intensities in the control (only vehicle treatment) and test animals (animals treated for 27 days with two different natural molecules belonging to the flavonoid family). The b-wave amplitude recorded from the animals of both treatment groups (naringenin and quercetin) shows a significant increase compared to the control group (**p = 0.007; **p = 0.01, respectively). In addition, the kinetics of ERG responses to the brightest stimulus, are faster in the animals treated with either one of the two molecules compared to the control (*p = 0.045; *p = 0.014, respectively) (Figures 1B,C). Furthermore, the prolonged treatment up to P60 with naringenin shows persistence improved photopic ERG responses, which are otherwise almost extinct in the animals treated with the vehicle alone (Supplementary Figure 1). Although the functional response in mice treated with nutraceutical molecules is significantly increased compared to rd10 treated with vehicle, responses do not reach the levels of the wt mice, indicating only a partial recovery (Supplementary Figures 2C–E).

**FIGURE 2:**
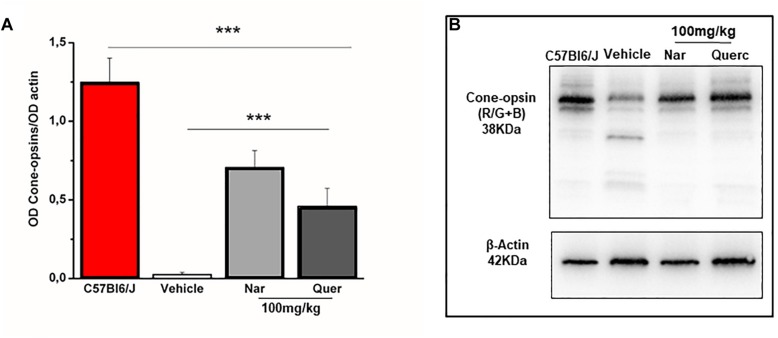
Naringenin and quercetin reduce the loss of cone after 27 days of treatment in rd10 mice. **(A)** Relative intensity of total cone-opsins (R/G + B) immunoreactive band quantified by densitometry scanning. Asterisks indicate significance (***p < 0.001) by two-way ANOVA followed by pair-wise *t*-test comparison with Bonferroni’s correction (*n* = 4 for wt C57Bl/6J mice and *n* = 6 for each treatment group). **(B)** Scanning image to representative immunoblots.

**FIGURE 3:**
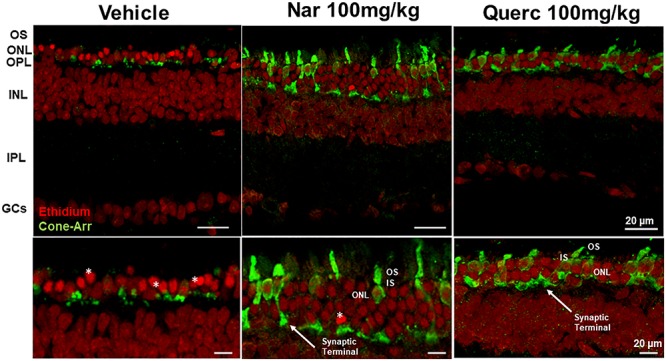
Morphological effects of chronic treatment with naringenin and quercetin on the outer retina. Immunohistochemistry for cones (cone-arrestin in green) and nuclear layers (Ethidium bromide in red). Cone-arrestin staining revealed the protective effect of chronic treatment of both nutraceutical compounds on degeneration of cone photoreceptors. OS, outer photoreceptor segments; ONL, outer nuclear layer; OPL, outer plexiform layer; INL, inner nuclear layer; IPL, inner plexiform layer; GCs, ganglion cells; *, pyknotic nuclei.

To assess whether the observed recovery of retinal function is due to an increased cone survival, we performed the quantification of both cone-opsins (middle-wavelength and short-wavelength cone opsins) by western blot, in order to assess the total content of these proteins (Figure 2). Figure 2A shows that despite cone-opsin protein levels being reduced in all rd10 groups compared to the wt control group (***p < 0.001) there is a significant increase in cone-opsins protein content after chronic treatment with natural molecules compared to rd10 mice treated with vehicle alone (***p < 0.001). Moreover, the immunohistochemistry for cone-arrestin (Figure 3, green staining) shows that the retinas taken from the animals treated with naringenin or quercetin, present a complete cone layer in the ONL, while in the retinas of animals treated with the vehicle alone, only a sporadic and delocalized label is observed, indicating the progressive degeneration of the cone. Figure 3 shows that preservation of an intact morphological structure (including outer/inner photoreceptor segments (OS/IS) and terminal pedicle) similar to wt cones (Supplementary Figure 2A) is achieved in some but not all cones. The remaining cone photoreceptors have an OS only partially preserved and their nuclei are in a pre-apoptotic phase (bright red of ethidium). These morphological alterations can be the structural correlate of the recovered, but still below wt level, light responses measured by ERG in mice treated with nutraceutical molecules.

### Naringenin and Quercetin Are Effective in Reducing Oxidative Stress in the Cellular Environment

After observing an improvement in both functional response and retinal morphology in animals treated with naringenin or quercetin, we went on to evaluate the influence of nutraceutical treatments on the levels of oxidative stress in the cellular environment. The flavonoids could interact both with the enzymatic systems of cellular detoxification and through a scavenger’s action (Al-Dosari et al., 2017).

**FIGURE 4:**
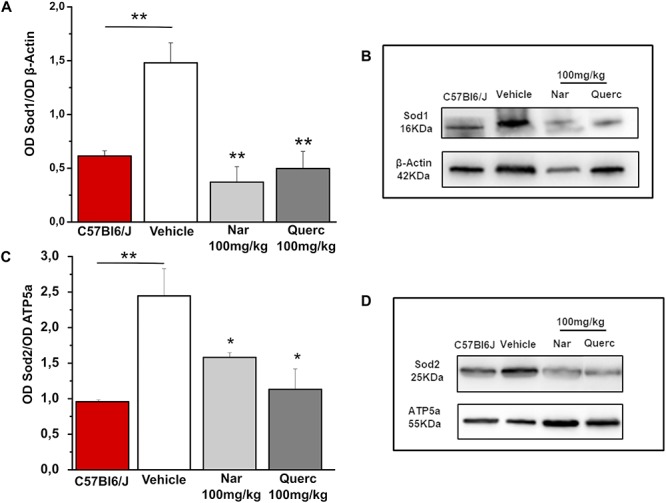
Chronic treatment with naringenin and quercetin significantly reduces the expression levels of anti-oxidant enzymes. **(A,C)** Relative intensity of Sod1 and Sod2 immunoreactive bands quantified by densitometry scanning, respectively (*n* = 3 for wt C57Bl/6J mice and *n* = 5 for each treatment group). Asterisks indicate significance (*p ≤ 0.05; **p ≤ 0.01) by one-way ANOVA with Bonferroni’s multiple comparison test. **(B,D)** Representative immunoblots of Sod1 and Sod2, respectively.

In order to understand if the nutraceutical treatment is able to induce a detoxifying response we evaluated the protein levels of Sod1 (cytosolic enzyme) and Sod2 (mitochondrial enzyme) that represent a major system of ROS detox of the internal milieu, by western blot analysis (Figure 4). Figure 4A shows a significant reduction of Sod1 expression levels in the naringenin-treated group and in the quercetin-treated group compared with the control group (**p = 0.008; **p = 0.003, respectively). In Figure 4C, the expression levels of Sod2 are significantly reduced in both groups of treated animals (naringenin and quercetin) compared to the group treated with the vehicle alone (*p = 0.037; *p = 0.021, respectively). Furthermore, it is possible to note that, as a result of both treatments performed, and for both proteins evaluated (Figures 4A,C), there is a return to the physiological conditions found in the age-matched wt animals (**p = 0.01 wt vs. rd10 untreated). These results indicate that ROS detoxification systems are reduced after nutraceutical treatment, probably because of a scavenger action of the flavonoids or a reduction of ROS production levels.

**FIGURE 5:**
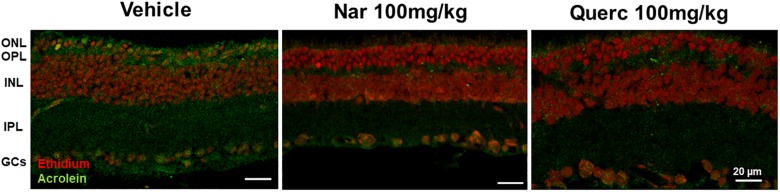
Lipid peroxidation in rd10 mouse retina. Immunolabeling of acrolein (green staining), a toxic product formed during lipid peroxidation and used as a marker of oxidative stress, is reduced after treatment with naringenin or quercetin for 27 days as compared with control. ONL, outer nuclear layer; OPL, outer plexiform layer; INL, inner nuclear layer; IPL, inner plexiform layer; GCs, ganglion cells.

To evaluate if the nutraceutical treatment induces a reduction of ROS in the retina, we used a lipid peroxidation marker, acrolein, and index of ROS levels that binds lipids of cell membranes (Komeima et al., 2006). Figure 5 shows how the marking for acrolein (green staining) is highly visible in the retinal sections obtained from animals treated with the vehicle while the green staining is reduced in the retinal sections obtained from both treatment groups returning to the physiological condition present in the age-matched wt animals (Supplementary Figure 2B) suggesting a direct action of the two molecules as a scavenger.

## Discussion

A previous study conducted in our laboratory has shown that function and morphology of cones, in rd10 mice aged P45, are altered with respect to the wt animals of the same age (Piano et al., 2013) and other works have shown the predominant role of oxidative stress in the secondary death of cone-photoreceptors, following the primary death (mutation-dependent) of rod, in several RP models (Shen et al., 2005; Komeima et al., 2006). Further studies, conducted in several animal models of RP, have confirmed the strong involvement of oxidative stress in the degeneration of the cone. These studies have also shown how different therapeutic approaches, that target oxidative stress, are effective in slowing the progressive degeneration of the cone photoreceptors (Assimopoulou et al., 2005; Shen et al., 2005; Komeima et al., 2006, 2007, 2008; Kanakis et al., 2007; Fernandez-Sanchez et al., 2012; Xiong et al., 2015; Wang et al., 2016; Trouillet et al., 2018).

In the present study, we tested a novel therapeutic strategy using two flavonoids, naringenin and quercetin, to limit the oxidative stress underlying the RP. Indeed, these flavonoids exhibit anti-oxidant properties interacting with enzymatic system of cellular detoxification through a scavenger’s action (Al-Dosari et al., 2017). Furthermore, it has been demonstrated that the anti-oxidant and anti-apoptotic effects of flavonoids may limit neurodegeneration by providing neurotrophic support to prevent retinal damage in RP like in other kind of retinal disorders such as diabetic retinopathy (Al-Dosari et al., 2017) and Age-Related Macular Degeneration (Gopinath et al., 2018).

Our results confirm, and extend, the previous data published by other groups on the fundamental role of the increase of the oxidative stress in the death of cone photoreceptors and the beneficial effects of anti-oxidant molecules in slowing the degeneration of cone (Assimopoulou et al., 2005; Shen et al., 2005; Komeima et al., 2006, 2007, 2008; Kanakis et al., 2007; Fernandez-Sanchez et al., 2012; Xiong et al., 2015; Wang et al., 2016; Trouillet et al., 2018). In particular, we observed that a chronic non-invasive treatment with molecules of natural origin, frequently present in normal daily diets, can significantly slow down the progression of the disease. This reduction of oxidative damage resulted in increased survival of cone photoreceptors at P45, proving that oxidative damage contributes to cone cell death in the rd10 mouse model of RP. For instance, it has also been reported that over-expression of Sod1 and 2 may cause an aggravation of the oxidative damage and precipitate cone death in different RP models (Usui et al., 2009, 2011). The hypothesis that both nutraceutical compounds could slow down the cone degeneration is also supported by the demonstration that antioxidant-treated rd10 mice had a significant increase in ERG photopic b-wave amplitude and a reduction of implicit time, at P45 compared to vehicle-treated mice.

The importance of the detoxifying enzymes Sod1 and Sod2 has already been demonstrated in the rd10 mouse model of RP (Usui et al., 2011). Surprisingly, oral treatment, started at P18, with two molecules belonging to the flavonoid family, does not seem to interact with ROS detoxification systems, since the protein levels of Sod1 and Sod2 were lower than in the control group (vehicle alone). Therefore, our data suggest that naringenin and quercetin are able to interfere with the decrease of ROS levels in the cellular environment, through a different pathway.

As a consequence of the rod death, the oxygen consumption is reduced since the choroidal vessels are incapable of autoregulation and continue to provide the same blood flow, leading to an increase in the oxygen radical species, which in being highly reactive, attack the cellular structures (proteins, lipids, and nucleic acids) (Campochiaro and Mir, 2018). In particular, when free radicals attack double bonds in lipids they generate acrolein, 4-hydroxynonenal, and its presence indicates that lipid peroxidation has occurred, and the measurement of their levels provides a quantitative assessment of the amount of damage (Benedetti et al., 1980; Esterbauer and Cheeseman, 1990; Esterbauer et al., 1991).

The data obtained by acrolein stained in the retinal sections from naringenin and quercetin treated-mice show how this specific marker of oxidative stress is almost completely absent compared to the animals treated with the vehicle alone. This result demonstrates that the chronic treatment with nutraceutical molecules is sufficient to reduce the ROS levels in the cellular environment. This effect could be due to a direct action between flavonoids and ROS or it could be due to an improvement of the cellular metabolic conditions (for example a better efficiency of cellular respiration at the mitochondria level) which leads to an upstream reduction in the production of ROS. Therefore, further experiments are required to deepen this aspect as a logical continuation of the ongoing research on this topic.

The anti-oxidant and anti-apoptotic effects of flavonoids may limit neurodegeneration by providing neurotrophic support to prevent retinal damage in RP like in other kind of retinal pathologies such as diabetic retinopathy (Al-Dosari et al., 2017).

Overall, in this paper we used the rd10 mouse model of RP for the first time, to test the potential beneficial effects of two nutraceutical compounds, naringenin and quercetin, present in a typical western diet by a chronic non-invasive treatment.

In conclusion, our results demonstrate that a supplemental daily diet with sufficient doses of flavonoids could be an effective, mutation-independent and non-invasive approach, to slow retinal degeneration. Further validation of this approach requires additional work to estimate the actual effectiveness of the treatment for vision and to establish the optimized regimen of exogenous antioxidant molecules.

## Ethics Statement

This study was carried out in accordance with the recommendations of DL March 04, 2014, number 26 which implemented the Directive number 2010/63/UE, Italian Ministry of Health. The protocol #DGSAF0001996/2014 was approved by the Ethical Committees of University of Pisa.

## Author Contributions

IP and VD conceived and performed the experiments. IP, VD, and CG collected and critically analyzed the data. IP and CG wrote the manuscript. LT and VC critically reviewed the manuscript.

## Conflict of Interest Statement

The authors declare that the research was conducted in the absence of any commercial or financial relationships that could be construed as a potential conflict of interest.

## Abbreviations

BSA: bovine serum albumin
ERG: electroretinogram
GCs: ganglion cells
INL: inner nuclear layer
IPL: inner plexiform layer
ONL: outer nuclear layer
OPL: outer plexiform layer
OS: outer photoreceptor segments
PBS: phosphate buffered saline
ROS: superoxide radicals
RP: retinitis pigmentosa
Sod: superoxide dismutase
TBS: Tris–buffered saline.
