# Supplementary material for: A Nutraceutical Strategy to Slowing Down the Progression of Cone Death in an Animal Model of Retinitis Pigmentosa
Source: Front Neurosci. 2019 May 17;13:461. doi: 10.3389/fnins.2019.00461 (PMC6533548; doi:10.3389/fnins.2019.00461)
Supplement: Supplementary file 1 [file Image_1.pdf]

Supplementary Material

Figure Supp.1

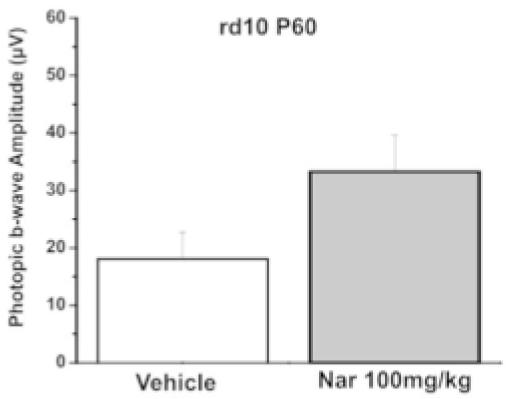

Supplementary 1. Naringenin slows down the decline of photoreceptor function in rd10 mice at P60. Photopic ERGs were performed a P60. The treatment with naringenin slow down, although not significantly, the decline of photoreceptor function in rd10 mice. Histograms represent the b-wave amplitude in response to the brightest flash. The bars are expressed as average ± SEM. The number of animals for each treatment group is n= 5.

Figure Supp. 2

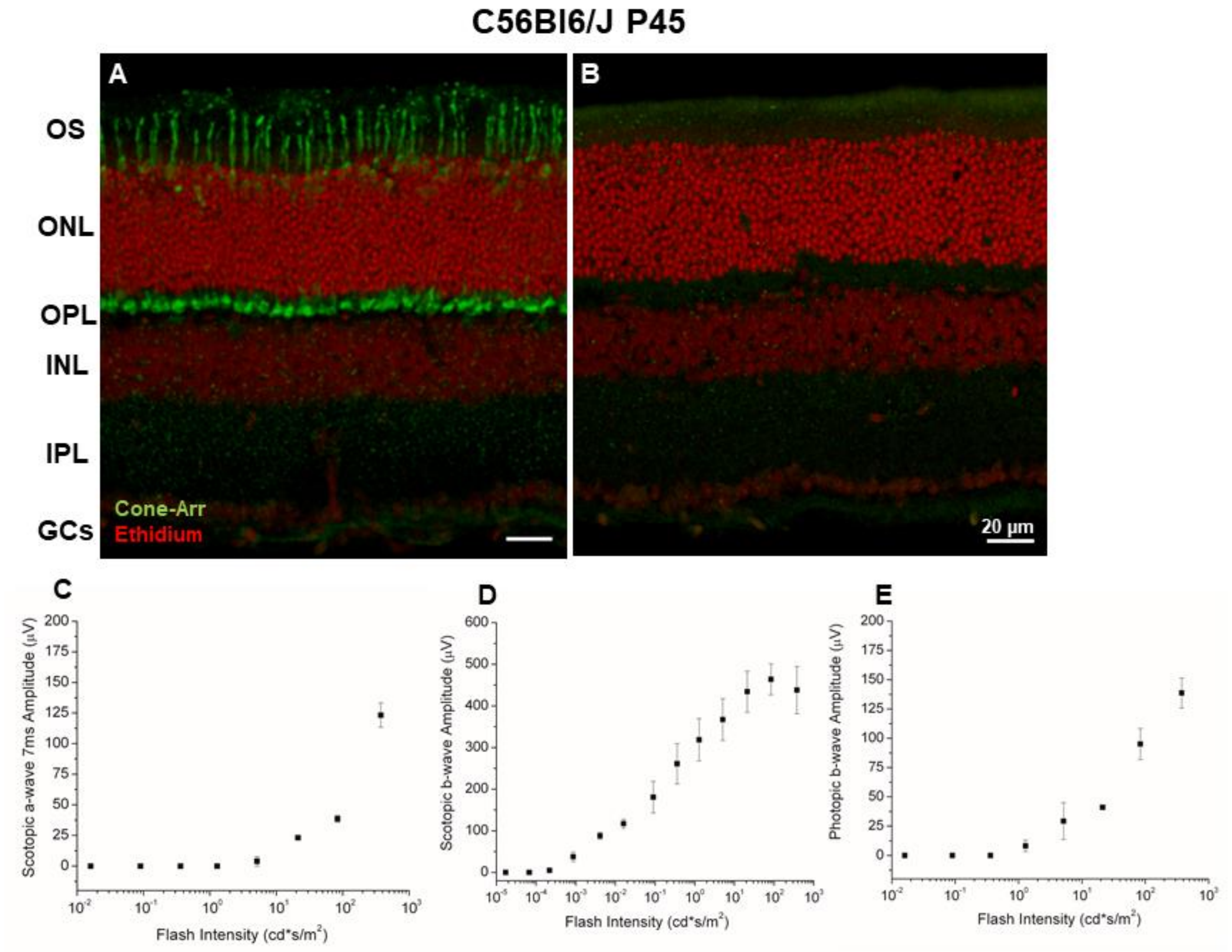

Supplementary 2. Morpho-functional analysis of the C57Bl6/J at P45. A) Immunohistochemistry for cone-arrestin (green staining) and nuclear layers (Ethidium, red). Cone show normal morphology. B) Immunohistochemistry for α-acrolein (green staining) and nuclear layers (Ethidium, red). Acrolein, a marker of lipid peroxidation, is not present in wild type C57Bl/6J mice. Abbreviations: OS: outer photoreceptor segments; ONL: outer nuclear layer; OPL: outer plexiform layer; INL: inner nuclear layer; IPL: inner plexiform layer; GCs: ganglion cells. C-E) Sensibility curve (amplitude as a function of different flash intensity) of both scotopic and photopic ERG recordings obtained from C57Bl6/J (n=4); value is expressed as average ± SEM.
